# Mechanisims of asthma and allergic disease – 1073. Image analyses and cell activation of Der p2 in human epithelium

**DOI:** 10.1186/1939-4551-6-S1-P70

**Published:** 2013-04-23

**Authors:** Jawji Tsai

**Affiliations:** 1 Department of Medical Research, Taichung Veterans General Hospital, Taiwan

## Background

House dust mites can cause airway inflammation. Its major allergen Der p2 causes inflammation with functions mimicking myeloid differentiation-2 (MD2). The epithelium activated by Der p2 and its relationship with LPS-promoted MD2/TLR signaling remained obscure.

## Objective

To provide image analyses of Der p2 and MD2 in epithelium.

## Methods

Both nasal polyps and BEAS-2B were used to examine the internalization of Der p2 in epithelium. The upregulation of MD2 was determined by messenger RNA (mRNA) and protein expression, and confirmed by amino acid sequencing. The cytokine secretions of Interleukin-6/Interleukin-8 (IL-6/IL-8) from epithelium were measured. The effects of Der p2 on BEAS-2B were further investigated by LPS-promoted MD2/TLR signaling and cytokine secretions, and evaluated by antibodies of TLR and inhibitors of transcription factor.

## Results

The expression of MD2 was increased in epithelium of nasal polyps and BEAS-2B after rDer p2 treatment. After co-immunoprecipitation with anti-Der p2, immuno-reactive MD2 could be identified. Der p2-EGFP could localize in endoplasmic reticulum (ER). In the presence of rDer p2, the secretions of IL-6/IL-8 by BEAS-2B were trivial but augmented by LPS and reduced by anti-MD2. When BEAS-2B was cultured with rDer p2 in conjunction with LPS, the mRNA expression of TLR2 and IL-6/IL-8 were increased. The increments were downregulated by Mitogen-Activated Protein Kinase (MAPK) inhibitors, dexamethasone, calcitriol and neutralizing antibody of TLR2.

## Conclusions

We provided evidences of Der p2 internalization and MD2 upregulation in epithelium. The synergistic effects of Der p2 and LPS on IL-6/IL-8 secretions were through TLR2/MAPK.MD2 upregulation could serve as an indicator for Der p2-induced airway inflammation.

